# The A930G Polymorphism of *P22phox* (CYBA) Gene but Not C242T Variation Is Associated with Hypertension: A Meta-Analysis

**DOI:** 10.1371/journal.pone.0082465

**Published:** 2013-12-09

**Authors:** Yu-Wang Qin, Jiao Peng, Bao-Yun Liang, Li Su, Qing Chen, Juan-Juan Xie, Lian Gu

**Affiliations:** 1 First Affiliated Hospital, Guangxi University of Chinese Medicine, Nanning, Guangxi, China; 2 Pre-Clinical Faculty of Guangxi Medical University, Nanning, Guangxi, China; 3 School of Public Health of Guangxi Medical University, Nanning, Guangxi, China; Baylor College of Medicine, United States of America

## Abstract

**Background:**

Recently, it has been reported that the A930G and C242T polymorphisms within p22phox (CYBA) gene are involved in the pathogenesis of hypertension. However, the results remain controversial. Furthermore, no previous meta-analysis has been conducted to evaluate the relationship between the A930G and C242T polymorphisms and hypertension. Therefore, we performed this meta-analysis to clarify these controversies.

**Objective and Methods:**

All of the included articles were retrieved from the PubMed and Embase databases, as well as the CNKI, CBM, Chongqing VIP and Wan Fang databases according to the Preferred Reporting Items for Systematic Reviews and Meta-Analyses (PRISMA) guidelines. Odds ratios (OR) with corresponding 95% confidence intervals (CI) were used to assess the strength of the association. Accounting for heterogeneity, a fixed or random effects model was respectively adopted. Heterogeneity was checked using the Q test and the I^2^ statistic. A cumulative meta-analysis was conducted to estimate the tendency of pooled OR. Funnel plots and Egger’s tests were performed to test for possible publication bias.

**Results:**

Five articles on A930G with 2003 cases/2434 controls and eight articles on C242T with 2644 cases/1967 controls were identiﬁed. A signiﬁcant association of A930G polymorphisms with the risk of hypertension was found in the dominant model (OR=0.59, 95% CI: 0.38–0.92, p=0.021) and allelic model (OR=0.66, 95% CI: 0.46–0.95, p=0.024). In the stratified analysis, a signiﬁcant association could be found among the hospital-based and population-based studies. However, no evidence of a significant association of the C242T polymorphism with hypertension was found in the overall analysis and subgroup analysis.

**Conclusions:**

This meta-analysis indicates that the A930G polymorphism, but not the C242T variation, might be a protective factor for hypertension.

## Introduction

Hypertension is now considered a major public health issue [1] that affects nearly one billion people worldwide [2]. It is recognized as the leading contributor to death and disability globally [3], and the prevalence is dramatically increasing [4,5]. According to the World Health Organization (WHO), hypertension can be attributed to the loss of 7.6 million lives annually (13.5% of all deaths globally) and the loss of 57 million disability-adjusted life years (DALYs) worldwide [6]. Hypertension is one of the primary risk factors for cardiovascular disease [7], such as stroke [8-10] and coronary heart disease [11]. Nearly 54% of stroke and 47% of coronary heart disease cases can be attributed to hypertension [7]. Hypertension is widely accepted as a multifactorial disease, resulting from the interaction of many risk genes together with environmental factors [12]. Approximately 30% to 50% of the variation of blood pressure in the general population is genetically determined [13].

The p22phox (CYBA) gene is located on the long arm of chromosome 16q24 [14-16]. It encodes for p22phox, a major component of nicotinamide adenine dinucleotide phosphate (NADPH) oxidases and plays a crucial role in the activation of NADPH oxidase [17-20]. The NADPH oxidase system, which constitutes the most important source of reactive oxygen species (ROS) in the vessel wall, is mostly expressed in phagocytes, endothelial cells, smooth muscle cells and ﬁbroblasts [21,22]. ROS induce oxidative stress, and have been implicated in the pathogenesis of hypertension [23-25]. An animal study showed that functional polymorphisms in the p22phox gene promoter are associated with hypertension [26]. Several functional polymorphisms in the p22phox gene have also been explored in association with hypertension. The p22phox gene A675T polymorphism plays a functional role in NADPH oxidase-mediated oxidative stress in patients that suffer from hypertension [27]. However, the *CYBA* C852G and C536T polymorphisms have not been reported in association with hypertension [27]. 

Recently, more studies have focused on the association of the *CYBA* A930G and C242T polymorphisms with hypertension. One study conducted by Sales et al. [28] indicated that the A930G polymorphism is not related to hypertension. However, Kokubo et al. [29], Moreno et al. [30], Pang et al. [31] and Ha et al. [32] support the association between the A930G polymorphism and hypertension. Similarly, a statistically significant association between the C242T polymorphism and hypertension has been reported by Ji et al. [33]. Subsequent studies [34-40] have been conducted to investigate the association between the C242T polymorphism and hypertension. However, the results have generated considerable controversy. To the best of our knowledge, no meta-analyses on the association between the A930G and C242T polymorphisms and hypertension have been conducted. Therefore, we performed this meta-analysis to better clarify the association between the A930G and C242T polymorphisms and hypertension in view of the abovementioned inconsistent results. The association was also evaluated by further subgroup analysis according to ethnicity, the sources of controls and Hardy-Weinberg equilibrium (HWE).

## Materials and Methods

### Search strategy

In accordance with the Preferred Reporting Items for Systematic Reviews and Meta-Analyses (PRISMA) guidelines [41], we searched electronic databases including PubMed, Embase, Chinese National Knowledge Infrastructure (CNKI), Chinese Biological Medical Literature database (CBM), Wan Fang and Chongqing VIP database with no language restrictions. The literature search was updated on July 1, 2013 using the following keywords: ("A930G" OR "C242T" OR “p22phox” OR “CYBA” OR “NADPH oxidase” OR “nicotinamide adenine dinucleotide phosphate oxidase”) AND (“polymorphism” OR “mut*” OR “varia*”) AND ("high blood pressure" OR "hypertension" OR "arterial hypertension" OR "hypertensive disorder"). In order to acquire the relevant publications, reference lists of all retrieved publications were also scanned. We contacted the authors by email to request detailed information if necessary.

### Selection criteria

All the included original studies were selected according to the following inclusion criteria: a) studies evaluating the association between *CYBA* A930G or C242T polymorphisms and hypertension; b) case-control or cohort studies; c) sufficient data provided with information on the genotypes and allele frequencies. The exclusion criteria were: a) pedigree and family-based studies, b) duplicate publications. 

### Data extraction

The data were carefully extracted from all the included publications independently by two investigators (Liang and Peng) according to the selection criteria listed above. If there was any disagreement, it was discussed among the authors or by consulting another reviewer (Su) to reach a consensus. Data extracted from the studies included the name of the ﬁrst author, publication year, country, ethnicity, study design, source of controls, number of cases and controls, diagnostic criteria and selection criteria of cases and controls, genotyping methods, genotype or allele distribution, and the matching method. Incomplete data regarding the genotypes and allele frequencies were calculated using the available information.

### Statistical analysis

All statistical analyses were implemented in Stata statistical software version 11.1. The Hardy-Weinberg equilibrium (HWE) for the genotype distributions in controls was assessed by the chi-square test (p>0.05). We used the following genetic models to pool the data: dominant model (GG + GA versus AA/CC + CT versus TT), recessive model (GG versus GA + AA/CC versus CT +TT), codominant model 1 (GG versus GA/CC versus CT), codominant model 2 (GA versus AA/CT versus TT), and the allelic model (G allele versus A allele/C allele versus T allele). The strength of the association between the *CYBA* A930G or C242T polymorphism and hypertension was estimated by calculating ORs with corresponding 95% CIs. Heterogeneity was analyzed using the Q test and the I^2^ statistic. If significant heterogeneity existed (P<0.10, or I^2^ >50%), the random effects model was used to calculate the pooled OR [42]; otherwise, a fixed effects model was adopted [43,44]. Sensitivity analysis was performed to assess the stability of the results. Begg's test and Egger's test were performed to estimate the possible publication bias. The asymmetrical funnel plot and P<0.05 were considered representative of publication bias. To further detect heterogeneity, subgroup analyses were performed regarding ethnicity, the source of controls, and HWE. We also conducted a cumulative meta-analysis of the association between the *CYBA* C242T polymorphism and hypertension to assess the trends in the pooled OR over time under the dominant contrast in the random effect model. Studies included in the cumulative meta-analysis were sorted by the year of publication.

## Results

### Search results

**Figure 1:**
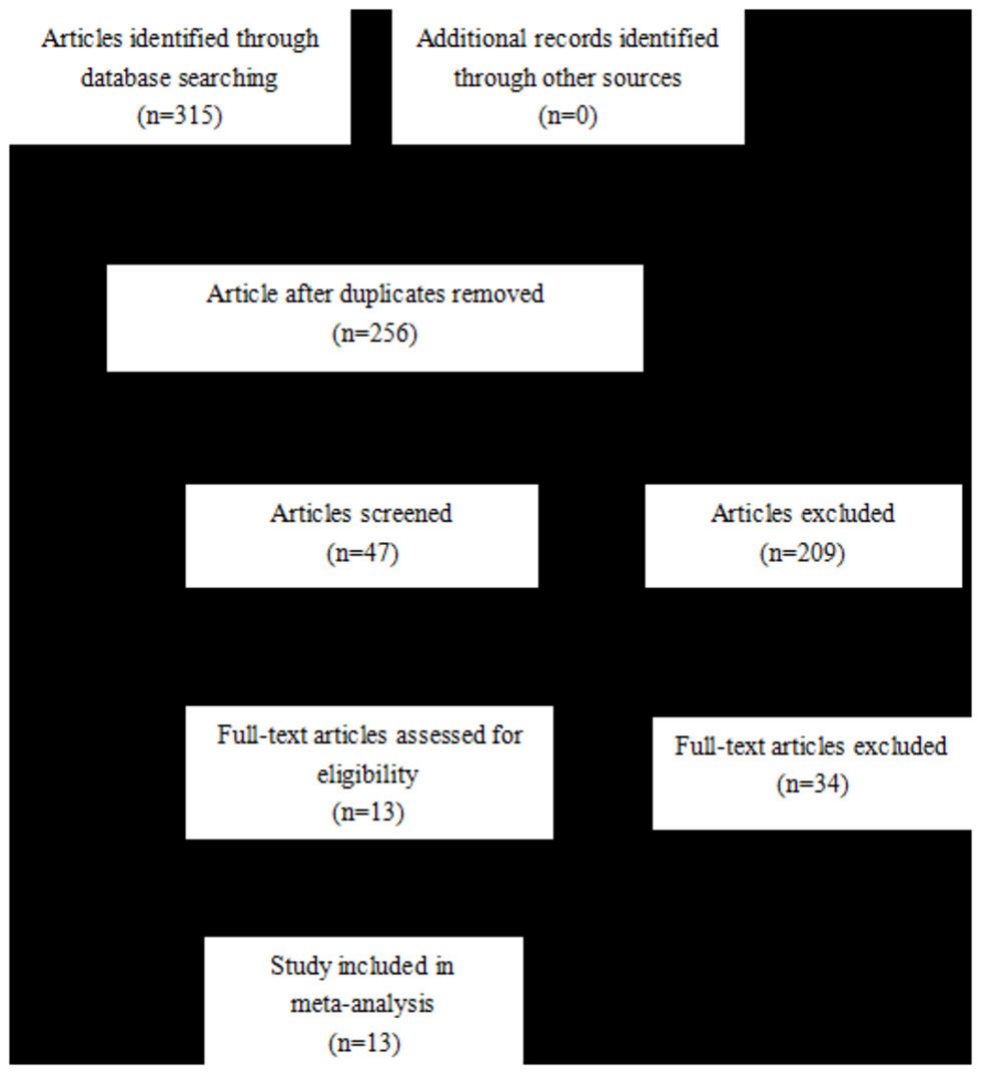
Flow chart of the articles selection for associations of the A930G/C242T polymorphism with hypertension. (Follow the PRISMA guidelines).

We identified 315 potentially relevant articles by our predeﬁned search strategy in the database of PubMed (n=48), Embase (n=67), CNKI (n=72), CBM (n=7), Chinese Wan Fang (n=62), and Chongqing VIP (n=22). After reviewing these titles and abstracts, we obtained 47 potential eligible studies. By scanning the full texts, 34 articles were excluded according to the selection criteria. Finally, thirteen qualiﬁed articles (five articles for A930G and eight articles for C242T) were included in the meta-analysis (Figure 1).

### Study characteristics

Five included studies investigating A930G polymorphisms in hypertension were examined, including 2003 cases and 2434 controls. All of the included studies were published between 2003 and 2007. The ethnicities of the research populations were as follows: three studies involved Asians and two involved in Caucasians. There were two population-based studies [28,29] and three hospital-based studies [30-32]. Polymerase chain reaction (PCR) was used for genotyping in all the included studies. The diagnostic criteria for hypertension were different among the studies: patients in three studies were diagnosed according to systolic blood pressure(SBP) and/or diastolic blood pressure (DBP) greater than 140 and 90 mmHg, patients in one study were diagnosed according to SBP and/or DBP greater than 139 and 89 mmHg, and the diagnostic criteria were not mentioned in one study. Controls were matched for gender, age, smokers, and other indices reported in three studies [30-32], but this was not mentioned in the other two studies [28,29]. The distribution of the genotypes in all the control groups obeyed HWE. The characteristics, genotypes, and allele frequencies are listed in Table 1. 

**Table 1:** The main characteristics of the eligible studies regarding associations between the *CYBA* A930G polymorphism and hypertension.

Authos	Ethnicity	Sample size		Diagnostic criteria		Matched	Genotyping methods	Source of controls	case					control					P value for HWE
		Cases	Controls	Cases	Controls				GG	AG	AA	G	A	GG	AG	AA	G	A	
Moreno et al./2003	Caucasian	88	68	SBP>139 mmHg/ DBP>89 mm Hg	normal blood pressure	gender, smokers, diabetes, GLU, TC, HDL-C, LDL-C	PCR	HB	36	41	11	113	63	23	29	16	75	61	0.3252
Ha et al./2004	Asian	83	66	SBP≥ 140 mmHg/ DBP≥ 90 mmHg	normal blood pressure	age, BMI, blood lipid levels	PCR	HB	35	38	10	108	58	19	26	21	64	68	0.0884
Pang et al./2004	Asian	123	105	SBP≥ 140 mmHg/ DBP≥ 90 mmHg	normal blood pressure	gender, age, GLU, HDL-C, LDL-C	PCR	HB	45	57	21	147	99	12	51	42	75	135	0.672
Kokubo et al./2005	Asian	1515	2125	SBP≥ 140 mmHg/ DBP≥ 90 mmHg	normal blood pressure	NA	PCR	PB	481	749	285	1711	1319	615	1050	460	2280	1970	0.7603
Sales et al./2007	Caucasian	194	70	NA	normal blood pressure	NA	PCR	PB	73	83	38	229	159	20	42	8	82	58	0.083

HWE: Hardy-Weinberg equilibrium; NA: not available; PCR: polymerase chain reaction; HB: hospital based; PB: population based; SBP: systolic blood pressure; DBP: diastolic blood pressure

GLU: glucose; HDL-C: high-density lipoprotein cholesterol; LDL-C: low-density lipoprotein cholesterol; TC: total cholesterol

 As shown in Table 2, eight studies (1842 cases/1967 controls) on the association of the C242T polymorphism and hypertension were included. Studies were conducted from 2003 to 2013 among various ethnicities (four studies involved in Caucasians and four involved Asians). Three studies were population-based [34,35,37] and five were hospital-based studies [33,36,38-40]. Genotyping was consistently performed in these studies by PCR. Patients with hypertension in five studies were diagnosed based on systolic and/or diastolic blood pressure over 140 and 90 mmHg. Among the other three studies, patients in one study were diagnosed according to systolic and/or diastolic blood pressure over 139 and 89 mmHg, patients in one study were diagnosed according to systolic and/or diastolic blood pressure over 130 and 80mmHg, and the diagnostic criteria were not mentioned in one study. Among the eight eligible studies, five studies [34,36,38-40] stated that the controls were matched for age, gender and clinical index; however, matching was not mentioned in the other three studies [33,35,37]. The distribution of genotypes and allele in the control group deviated from HWE in two studies. The general characteristics and the distribution of C242T genotypes and alleles in this meta-analysis are shown in Table 2.

**Table 2:** The main characteristics of the eligible studies regarding associations between the *CYBA* C242T polymorphism and hypertension.

Authors	Ethnicity	Sample size		Diagnostic criteria		Matched	Genotyping methods	Source of controls	case					control					P value for HWE
		Cases	Controls	Cases	Controls				CC	CT	TT	C	T	CC	CT	TT	C	T	
Ji et al./2003	Asian	57	106	SBP≥ 140 mmHg/ DBP≥ 90 mmHg	normal blood pressure	NA	PCR	HB	41	15	1	97	17	91	13	2	195	17	0.1251
Pang et al./2005	Asian	123	105	SBP≥ 140 mmHg/ DBP≥ 90 mmHg	normal blood pressure	age, gender, GLU, HDL-C, LDL-C	PCR	HB	89	29	5	207	39	91	13	1	195	15	0.4158
Hsueh et al./2005	Asian	79	213	SBP≥ 140 mmHg/ DBP≥ 90 mmHg	normal blood pressure	NA	PCR	PB	68	9	2	145	13	193	17	3	403	23	0.0156
Moreno et al./2006	Caucasian	326	297	SBP>139 mmHg/ DBP>89 mm Hg	normal blood pressure	gender, DM, TC, HDL-C, LDL-C	PCR	HB	133	143	50	409	243	93	156	48	342	252	0.2348
Wang et al./2007	Asian	135	135	SBP≥140mmHg/ DBP≥90 mmHg	SBP< 140/DBP< 90mmHg	age, gender	PCR	HB	113	22	0	248	22	100	35	0	235	35	0.1267
Kuznetsova et al./2008	Caucasian	272	97	NA	NA	NA	PCR	PB	131	122	19	384	160	41	52	4	134	60	0.0167
Schreiber et al./2012	Caucasian	1030	826	SBP≥ 140 mmHg/ DBP≥ 90 mmHg	normal blood pressure	age, gender, smokers, BMI, GLU, DM, LDL-C,HDL-C, triglycerides, uric acid	PCR	PB	452	459	119	1363	697	367	369	90	1103	549	0.8757
Petrovic et al./2013	Caucasian	622	188	Subjects with type 2 diabetes with SBP≥130mmHg or DBP ≥80mmHg	WHO Classification of Diabetes Mellitus	gender, DM, smokers, HbaIc, GLU, TC, LDL-C, HDL-C, triglycerides	PCR	HB	274	257	91	805	439	64	99	25	227	149	0.2221

HWE: Hardy-Weinberg equilibrium; NA: not available; PCR: polymerase chain reaction;HB: hospital based; PB: population based;SBP: systolic blood pressure; DBP: diastolic blood pressure

GLU: glucose; HDL-C: high-density lipoprotein cholesterol; LDL-C: low-density lipoprotein cholesterol; TC: total cholesterol

DM: diabetes mellitus; BMI: Body Mass Index

WHO: World Health Organization

### The A930G polymorphism associated with hypertension

**Figure 2:**
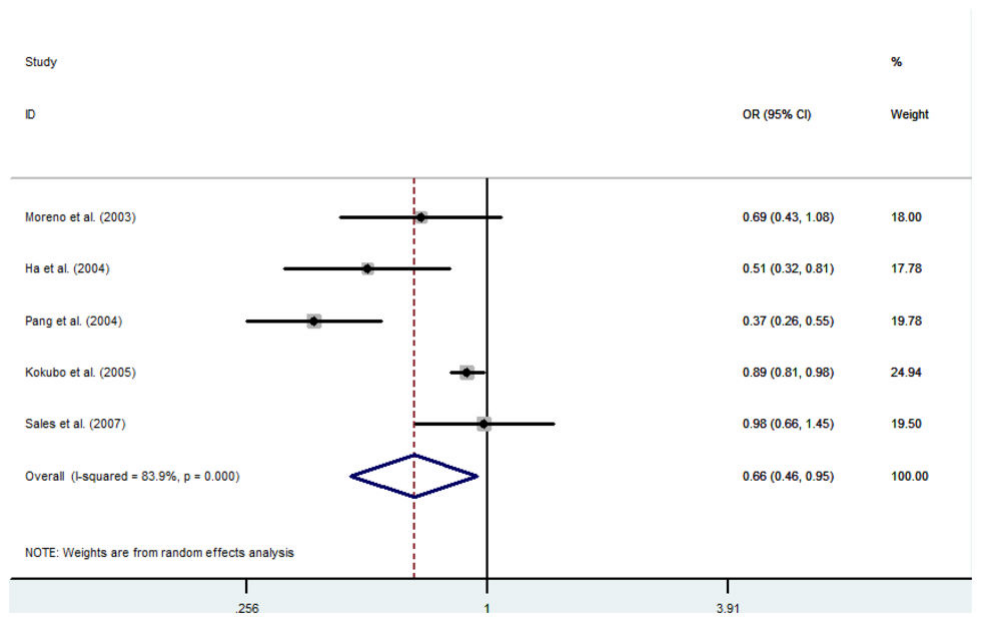
Forest plots of hypertension associated with the CYBA A930G polymorphism.

Significant associations between the A930G polymorphism and the risk of hypertension were identified in the dominant model (OR=0.59, 95%CI: 0.38-0.92, p=0.021) and the allelic model (OR=0.66, 95% CI: 0.46-0.95, p=0.024) (Figure 2). However, there was not a significant association in the other genetic models, i.e. the recessive model (OR=0.59, 95%CI: 0.32-1.07, p=0.083), co-dominant model 1 (OR=0.70, 95%CI: 0.40-1.21, p=0.197), or co-dominant model 2 (OR=0.68, 95%CI: 0.46-1.01, p=0.054) (Table 3).

**Table 3:** Main Results of ORs with 95%CI of CYBA A930G polymorphism and hypertension.

**Variables**		**No.**	**Dominant model**		**Recessive model**		**Codominant model 1**		**Codominant model 2**		**Allele model**	
			**OR (95%CI)**	**P**	**OR (95%CI)**	**P**	**OR (95%CI)**	**P**	**OR (95%CI)**	**P**	**OR (95%CI)**	**P**
**Total**		5	0.59(0.38-0.92)	0.021	0.59(0.32-1.07)	0.083	0.70(0.40-1.21)	0.197	0.68(0.46-1.01)	0.054	0.66(0.46-0.95)	0.024
**Ethnicity**	**Caucasian**	2	0.70(0.45-1.08)	0.107	0.94(0.24-3.72)	0.930	1.09(0.23-5.22)	0.914	0.68(0.41-1.12)	0.127	0.84(0.59-1.19)	0.318
	**Asian**	3	0.50(0.22-1.13)	0.098	0.45(0.20-1.02)	0.056	0.56(0.30-1.05)	0.071	0.64(0.33-1.23)	0.178	0.57(0.31-1.04)	0.067
**Source of controls**	**HB**	3	0.45(0.23-0.91)	0.027	0.34(0.22-0.52)	0.000	0.42(0.27-0.66)	0.000	0.60(0.30-1.19)	0.144	0.50(0.35-0.71)	0.000
	**PB**	2	0.86(0.75-0.99)	0.037	1.14(0.53-2.45)	0.746	1.32(0.50-3.53)	0.576	0.77(0.48-1.24)	0.283	0.90(0.82-0.98)	0.019

**Figure 3:**
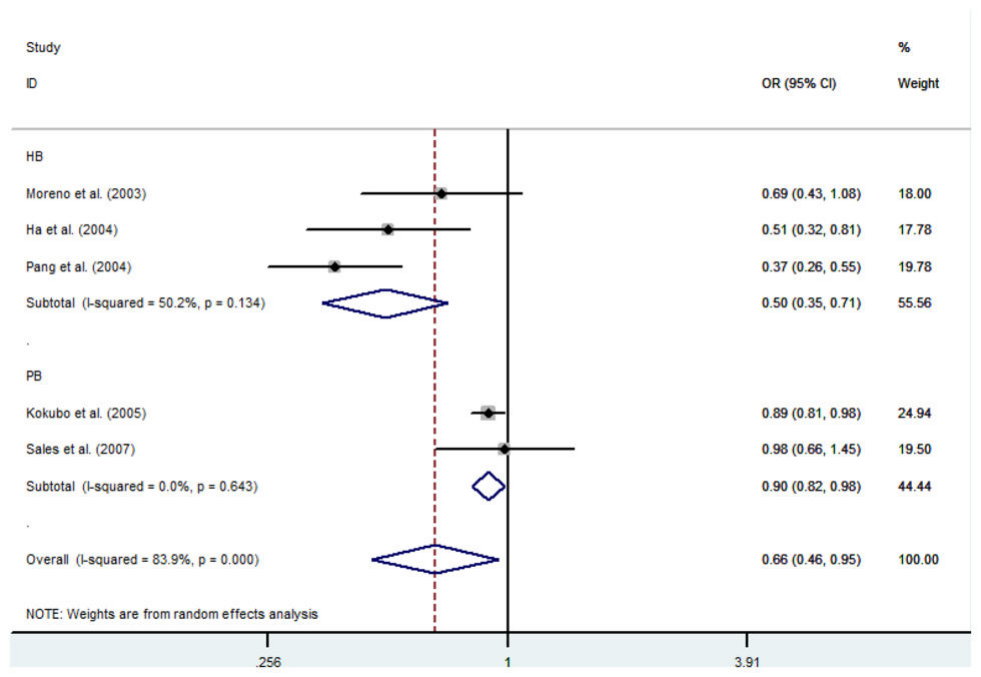
Forest plots for the association of the CYBA A930G polymorphism in the stratified analysis for source of controls.

In the further subgroup analysis according to the source of controls, a statistically significant association of the A930G polymorphism with hypertension was found in the dominant model in the hospital-based studies (OR: 0.45, 95%CI: 0.23-0.91, p=0.027) and the population-based studies (OR: 0.86, 95%CI: 0.75-0.99, p=0.037). Similar results were found in the allelic model among the hospital-based studies (OR: 0.50, 95%CI: 0.35-0.71, p=0.000) and the population-based studies (OR: 0.90, 95%CI: 0.82-0.98, p=0.019) (Figure 3). Statistical signiﬁcance was also found in the recessive model (OR: 0.34, 95%CI: 0.22-0.52, p=0.000) and co-dominant model 1 (OR: 0.42 95%CI: 0.27-0.66, p=0.000) in the hospital-based studies, but not in the population-based studies (recessive model: OR=1.14, 95%CI: 0.53-2.45, p=0.746; OR=1.32 95%CI:0.50-3.53, p=0.576). However, no significance was observed in the co-dominant model 2 in either the hospital-based studies (OR: 0.60 95%CI: 0.30-1.19, p=0.144) or in the population-based studies (OR: 0.77 95%CI: 0.48-1.24, p=0.283). In the stratified analysis for ethnicity, no significant association was detected in any of the genetic models (Table 3).

### The C242T polymorphism associated with hypertension

**Figure 4:**
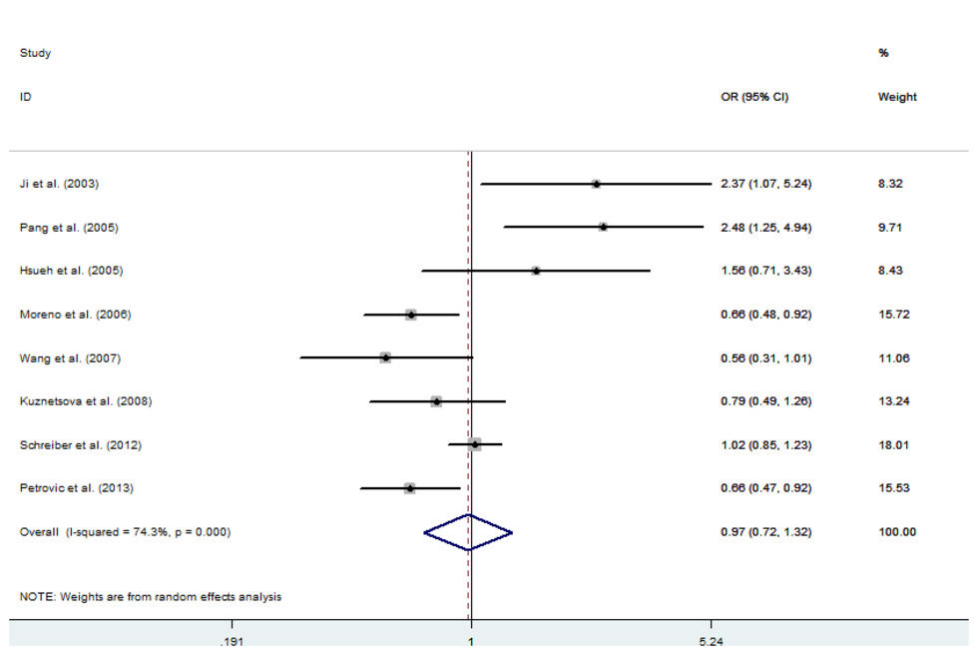
Forest plots of hypertension associated with the CYBA C242T polymorphism.

There was no statistically significant association between the C242T polymorphism and hypertension in any of the genetic models: dominant model (OR=0.97, 95% CI: 0.72-1.32, p=0.870), recessive model (OR=1.08, 95%CI: 0.88-1.34, p=0.443), co-dominant model 1 (OR=1.17, 95%CI: 0.94-1.46, p=0.162), co-dominant model 2 (OR=0.94, 95%CI: 0.68-1.29, p=0.692), and allelic model (OR=1.02, 95%CI:0.82-1.26, p=0.846) (Figure 4). Similarly, no significant association was detected between the C242T polymorphism and hypertension in any of the genetic models in the subgroup analysis. The results of this meta-analysis are listed in Table 4.

**Table 4:** Main results of ORs with 95%CI of CYBA C242T polymorphism and hypertension.

**Variables**		**No.**	**Dominant model**		**Recessive model**		**Codominant model 1**		**Codominant model 2**		**Allele model**	
			**OR (95%CI)**	**P**	**OR (95%CI)**	**P**	**OR (95%CI)**	**P**	**OR (95%CI)**	**P**	**OR (95%CI)**	**P**
		8	0.97(0.72-1.32)	0.870	1.08(0.88-1.34)	0.443	1.17(0.94-1.46)	0.162	0.94(0.68-1.29)	0.692	1.02(0.82-1.26)	0.846
**Ethnicity**	**Asian**	4	1.47(0.69-3.13)	0.317	2.03(0.61-6.75)	0.250	1.15(0.32-4.12)	0.827	1.44(0.67-3.10)	0.346	1.44(0.73-2.83)	0.288
	**Caucasian**	4	0.79(0.61-1.02)	0.076	1.06(0.86-1.31)	0.564	1.17(0.94-1.46)	0.167	0.75(0.56-1.00)	0.053	0.91(0.80-1.04)	0.187
**Source of controls**	**HB**	5	0.99(0.59-1.66)	0.974	1.05(0.76-1.43)	0.777	1.24(0.89-1.73)	0.196	0.96(0.57-1.62)	0.877	1.05(0.72-1.52)	0.808
	**PB**	3	1.00(0.81-1.24)	0.970	1.12(0.85-1.47)	0.439	1.11(0.83-1.49)	0.468	0.97(0.76-1.24)	0.818	1.03(0.91-1.17)	0.661
**HWE**	**YES**	6	0.97(0.67-1.40)	0.874	1.06(0.85-1.31)	0.604	1.14(0.91-1.43)	0.244	0.94(0.64-1.38)	0.764	1.01(0.78-1.31)	0.935
	**NO**	2	1.03(0.53-1.98)	0.934	1.77(0.69-4.53)	0.237	1.80(0.68-4.78)	0.238	0.96(0.49-1.89)	0.904	1.10(0.68-1.78)	0.691

### Sensitivity Analysis

Sensitivity analysis was performed to strengthen the confidence in the results by limiting the included studies according to HWE. After two studies [35,37] without HWE were excluded, the corresponding pooled ORs were not significantly altered. The results were accordance with those of the initial analysis. 

### Cumulative meta-analysis

**Figure 5:**
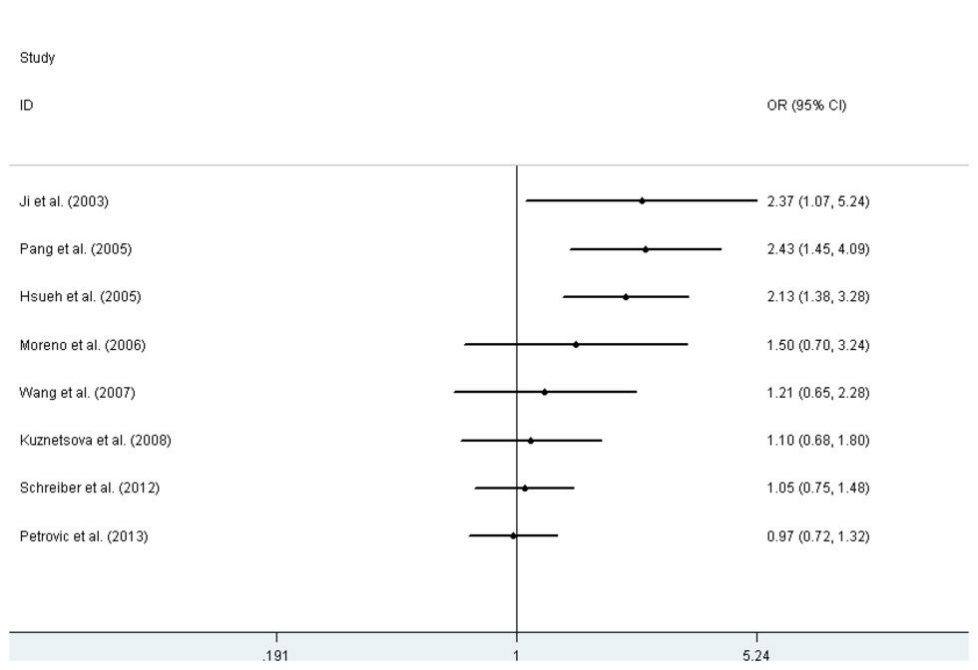
Cumulative plot of the association between the CYBA C242T polymorphism and hypertension for the dominate model chronologically.

Cumulative analysis of the C242T polymorphism with hypertension was performed according to the publication date. A signiﬁcant association was conﬁrmed in the dominant contrast among the studies from 2003 to 2005. However, subsequent publications from 2006 to 2013 failed to repeat the initial results: Moreno et al. (OR=1.50, 95%CI: 0.70-3.24), Wang et al. (OR=1.21, 95%CI: 0.65–2.28), Kuznetsova et al. (OR=1.10, 95%CI: 0.68–1.80), Schreiber et al. (OR=1.05, 95%CI: 0.75–1.48), Petrovicet al. (OR=0.97, 95%CI: 0.72–1.32) (Figure 5).

### Publication Bias

Funnel plots, Begg’s test and Egger's test were performed to assess the publication bias of the studies. The shapes of the funnel plots were visually symmetrical. No publication bias was found regarding the A930G and C242T polymorphism and its association with hypertension by using Egger’s test under the allelic model (t=-1.62, p=0.204/t=0.90, p=0.401), dominant model (t=-2.08, p=0.129/t=0.61, p=0.565), recessive model (t=-0.95, p=0.410/t=2.11, p=0.089), codominant model 1 (t=-0.63, p=0.574/t=0.72, p=0.501), or codominant model 2 (t=-1.71, p=0.186/t=0.56, p=0.597).

## Discussion

In this meta-analysis, thirteen qualiﬁed articles (five articles with 2003 cases/2434 controls for A930G and eight articles with 2644 cases/1967 controls for C242T) were included. To the best of our knowledge, this is the ﬁrst meta-analysis to assess the relationship of the A930G and C242T polymorphisms with hypertension. Our results suggest that the A930G polymorphism is associated with hypertension under both the dominant and allelic model, which was in accordance with the results of the studies by Kokubo et al. [29], Moreno et al. [30], and Pang et al. [31]. Large-scale studies are needed to further evaluate the association between the A930G polymorphism and hypertension. However, no significant of association between the C242T polymorphism and hypertension was observed in any genetic model, which was consistent with the ﬁndings of the studies by Schreiber et al. [34], Kuznetsova et al. [35] and Wang et al. [38]. In order to assess the robustness of our results, two studies were excluded for deviating from HWE, but the results of our meta-analysis were not altered, suggesting that our initial results were reliable. Even so, additional studies with larger sample sizes are required to further confirm the identified association.

In the stratified analysis by ethnicity, no significant association of the A930G polymorphism with hypertension was detected in any of the genetic models. Similarly, no significant association between the C242T polymorphism and hypertension could be found in any of the genetic models in the further subgroup analysis by ethnicity. According to the data from the International HapMap project, the frequency of the minor G allele in the A930G polymorphism (rs9932581) among the CEU population (northern and western European ancestry) is 0.350; the minor allele frequency (MAF) of the A930G polymorphism is similar among Japanese in Tokyo (JPT) (MAF=0.477), whereas it is A with a frequency of 0.360 among Han Chinese in Beijing (CHB). The MAF of the A930G polymorphism was found to be different between Caucasian populations and Asian Chinese populations according to the HapMap data. However, a sample size of 1515 cases and 2125 controls, which was used to evaluate the genetic susceptibility of hypertension in the Japanese population was larger than that in the Chinese population (206 cases/171 controls). The MAF of the A930G polymorphism is similar in Caucasian and Asian Japanese populations, which is in accordance to our findings that the effect of the A930G polymorphism on hypertension is similar across different ethnic populations. Furthermore, the C242T MAF varies between European and Asian populations, as the frequency of the T allele is higher in Europeans than in Asians (HapMap-CEU: 0.314, HapMap-CHB/JPT: 0.070). Our result is not consistent with the data from the International HapMap project, probably because of the limited eligible studies and sample sizes. Thus, large-scale studies are required to further evaluate our ﬁndings.

A significant association between the A930G polymorphism and hypertension was found for the dominant and allelic models in both the hospital-based studies and the population-based studies. However, a signiﬁcant association was found only in the hospital-based studies for the recessive model and co-dominant model 1, but not in the population-based studies, which might be attributed to differences in the sources of controls. The controls in hospital-based studies might suffer from other diseases that could possibly involve in the same genetic pathogenesis of hypertension. They may not be representative of the general population and result in a false positive. Thus, selective bias, which is likely to affect the quality and reliability of our findings, could not be ignored in the hospital-based studies. Therefore, this positive result should be interpreted with caution. As to the subgroup analysis of studies based on population, only two articles were included in the analysis; therefore, studies with larger sample sizes are required to further confirm our findings. Moreover, no significant association between the C242T polymorphism and hypertension could be found in any of the genetic models in the subgroup analysis according to the source of controls.

The biological mechanism of the A930G and C242T polymorphisms in the physiopathogenesis of hypertension is unclear. ROS have been suggested to play a major role in oxidative stress, and contribute significantly to the development of hypertension [24,25]. Higher production of ROS not only causes the inactivation of NO by an oxidative reaction [45,46], but also produces peroxynitrite [47]. Peroxynitrite mediates the oxidative inactivation of proteins, DNA, and lipids in the vascular endothelium [48], and leads to tissue injury. For this reason, ROS impair endothelial function [49] and significantly contribute to the development of hypertension [25]. 

According to the publication date, a cumulative analysis of the C242T polymorphism in hypertension was performed. A signiﬁcant association between the C242T polymorphism and hypertension was conﬁrmed with an increasingly narrow 95% confidence interval (CI) from 2003 to 2005. Nevertheless, no trend for the association between the C242T polymorphism and hypertension was found in subsequent studies from 2006 to 2013, which is in accordance with the results of the current meta-analysis. However, only eight studies were included in the cumulative analysis. To be sure of these results, further studies re required to conﬁrm our ﬁndings.

An exploration in the source of heterogeneity was conducted by subgroup analysis. Strong heterogeneity was still observed in studies assessing the relationship between the A930G polymorphism and hypertension when stratified by ethnicity. Thus, Asian and Caucasian ethnicity was likely to be one of the causes of heterogeneity. In addition, the environment that people lived in and genetic variations should be considered as sources of heterogeneity. Similarly, signiﬁcant heterogeneity still persisted in the subgroup analysis according to the source of controls, which suggested that the source of controls, whether studies were hospital-based or population-based, also had an influence on heterogeneity. Additionally, no obvious changes in heterogeneity could be observed among the studies of Asian populations in the association between the C242T polymorphism and hypertension when stratified by ethnicity. Nevertheless, heterogeneity decreased in studies on Caucasian populations, which indicated that the source of heterogeneity might originate from the Asian ethnicity. Heterogeneity was still signiﬁcant in hospital-based studies, but obviously reduced in the population-based studies, which suggested that heterogeneity might be attributed to the hospital-based studies. There was considerable variation in the sample sizes of the studies included in this meta-analysis; of the qualiﬁed studies on the A930G polymorphism in hypertension, the largest sample size was 1515 cases/2125 controls and the smallest sample size was 83 cases/66 controls. Similarly, of the included studies used to evaluate the association between the C242T polymorphism and genetic susceptibility to hypertension, the largest sample size was 1030 cases/826 controls and the smallest sample size was 57 cases/106 controls. These different sample sizes might also contribute to the source of heterogeneity. Furthermore, other confounding factors, such as matching methods, study design, and individual biological characteristics were identified as a potential source of heterogeneity. Furthermore, there was no publication bias in the present meta-analysis, as a relatively comprehensive search strategy was conducted. 

Finally, our results should be considered with some limitations in the present meta-analysis. First, the sample size in this meta-analysis was not large, which limited the statistical power. As to the subgroup analysis by ethnicity, only two Caucasian studies and three Asian studies were included for the A930G polymorphism in hypertension. Similarly, in the subgroup analysis according to the source of controls, only two population-based studies were included for the A930G polymorphism in hypertension, in contrast to three hospital-based studies. In order to better decipher our results, more studies with larger sample sizes are needed in the future. Second, this meta-analysis only focused on the articles from English and Chinese databases without language restrictions, which might lead to a potential language bias. Third, information on confounding factors, such as age, sex, smoking, and drinking, could not be obtained from all the original articles; these are considered effective inﬂuencing factors in the pathogenesis of hypertension. In addition, we did not perform an evaluation of potential interactions such as gene-gene or gene-environment, which might be involved in susceptibility to hypertension.

In summary, in the overall analysis, the present meta-analysis shows that the A930G polymorphism contributes to susceptibility to hypertension. Similarly, a statistically signiﬁcant association was found in the subgroup analysis according to ethnicity. However, there is a lack of evidence to support the association of C242T with hypertension. Further studies considering gene-gene and gene-environment interactions, larger sample sizes, and well-matched controls are needed to identify the association between the A930G and C242T polymorphisms and hypertension.

## Supporting Information

Checklist S1 **PRISMA checklist.**
(DOC)

File S1 **PRISMA 2009 Flow Diagram.**
(DOC)
