# Supplementary material for: The A930G Polymorphism of P22phox (CYBA) Gene but Not C242T Variation Is Associated with Hypertension: A Meta-Analysis
Source: PLoS One. 2013 Dec 9;8(12):e82465. doi: 10.1371/journal.pone.0082465 (PMC3857280; doi:10.1371/journal.pone.0082465)
Supplement: File S1 — PRISMA 2009 Flow Diagram. (DOC) [file pone.0082465.s002.doc]

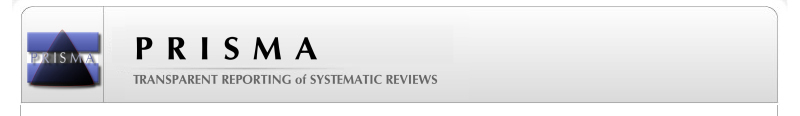
**PRISMA 2009 Flow Diagram**

**Screening**

**Included**

**Eligibility**

**Identification**

Records identified through database searching
(n = 315 )

Additional records identified through other sources
(n = 0 )

Records after duplicates removed
(n = 256 )

Records screened
(n = 47 )

Records excluded
(n = 209 )

Full-text articles assessed for eligibility
(n = 13 )

Full-text articles excluded, with reasons
(n = 34 )

Studies included in qualitative synthesis
(n = 13 )

Studies included in quantitative synthesis (meta-analysis)
(n =13 )
